# Supplementary material for: Thickness-dependent crack suppression and wrinkle formation in freestanding BaTiO3 membranes
Source: J Mater Res. 2026 Sep 3;41(19):3051–9. doi: 10.1557/s43578-026-01965-9 (PMC13743564; doi:10.1557/s43578-026-01965-9)
Supplement: Supplementary file 1 [file 43578_2026_1965_MOESM1_ESM.docx]

**Supplementary Information**

**Thickness-dependent crack suppression and wrinkle formation in freestanding BaTiO₃ membranes**

*Rajesh Mandal^*^, Ajay Kumar, Nini Pryds^*^*

*Department of Energy Conversion and Storage, Technical University of Denmark (DTU), Fysikvej, 310, 2800 Kgs. Lyngby, Denmark*

Email for correspondence: [***rajema@dtu.dk***](mailto:rajema@dtu.dk) ***,*** [***nipr@dtu.dk***](mailto:nipr@dtu.dk)

**Supplementary Figures:**

**SI 1:** Reciprocal Space Mapping (RSM) of (a) & (b) epitaxial BTO/SAO layer on STO for [103] and [002] planes, (c) BTO membrane transferred on Si/SiO_2_. (d) Rocking curve FWHM for the epitaxial BTO layer as well as the BTO membrane.

**SI 2:** Bright field large area image of the BTO membranes for the thickness ranges from 15 nm to 5 nm

**SI 3:** Bright field large area image of the BTO membranes for the support polymer CAB thickness ranges from 200 µm to 500 µm

**SI 4:** Bright field large area image as well as the AFM topography of the BTO membranes transferred on PDMS and gold coated silicon substrate

**SI 5:** Time evolution of the contrast ratio of the Amplitude and Phase for $\pm10$ V
